# Angiogenesis in NENs, with a focus on gastroenteropancreatic NENs: from biology to current and future therapeutic implications

**DOI:** 10.3389/fonc.2022.957068

**Published:** 2022-08-17

**Authors:** Eleonora Lauricella, Barbara Mandriani, Federica Cavallo, Gaetano Pezzicoli, Nada Chaoul, Camillo Porta, Mauro Cives

**Affiliations:** ^1^ Department of Interdisciplinary Medicine, University of Bari “Aldo Moro”, Bari, Italy; ^2^ Division of Medical Oncology, A.O.U. Consorziale Policlinico di Bari, Bari, Italy

**Keywords:** cabozantinib, lenvatinib, pazopanib, carcinoid tumor, TKIs (tyrosine kinase inhibitors)

## Abstract

Neuroendocrine neoplasms (NENs) are highly vascularized malignancies arising from cells of the diffuse neuroendocrine system. An intricated cross-talk exists between NEN cells and the tumor microenvironment, and three main molecular circuits (VEGF/VEGFR pathway, FGF-dependent signaling and PDGF/PDGFR axis) have been shown to regulate angiogenesis in these neoplasms. Multiple randomized trials have investigated antiangiogenic agents over the past two decades, and sunitinib is currently approved for the treatment of advanced, progressive, G1/G2 pancreatic NENs. In recent years, two phase III clinical trials have demonstrated the efficacy and safety of surufatinib, a multi-tyrosine kinase angioimmune inhibitor, in patients with well-differentiated pancreatic and extrapancreatic NENs, and two studies of this agent are currently underway in Europe and US. The HIF-2α inhibitor belzutifan has recently received regulatory approval for the treatment of tumors arising in the context of Von-Hippel Lindau syndrome including pancreatic NENs, and a study of this drug in patients with sporadic tumors is presently ongoing. Combinations of antiangiogenic agents with chemotherapeutics and targeted drugs have been tested, with accumulating toxicities being a matter of concern. The potential of antiangiogenic agents in fine-tuning the immune microenvironment of NENs to enhance the activity of immune checkpoint inhibitors has been only partially elucidated, and further research should be carried out at this regard. Here, we review the current understanding of the biology of angiogenesis in NENs and provide a summary of the latest clinical investigations on antiangiogenic drugs in this malignancy.

## Introduction

Neuroendocrine neoplasms (NENs) are heterogeneous malignancies arising from cells of the diffuse neuroendocrine system. They are often characterized by an indolent behavior and the ability to secrete a variety of peptide hormones and biogenic amines (1). The incidence of NENs has steadily increased in the last four decades, and NENs currently constitute the second most prevalent cancer of the gastroenteropancreatic (GEP) tract (2). According to the 2019 WHO classification (3), GEP-NENs can be subdivided in well-differentiated neuroendocrine tumors (NETs) and poorly differentiated neuroendocrine carcinomas (NECs). Neuroendocrine tumors can be further subdivided in low-grade (G1), intermediate-grade (G2) and high-grade (G3) tumors according to their proliferative activity, and large series have proven the prognostic relevance of such a classification (4, 5).

Neuroendocrine tumors are highly vascularized malignancies, and their intratumor vessel density is estimated to be approximately 10-fold higher than in carcinomas (6, 7). This feature is not particularly surprising, as it recapitulates the microscopic architecture of normal endocrine glands which are characterized by a dense vascular network facilitating hormone secretion. In this context, evidence demonstrates that the aberrant activation of the hypoxia-inducible factor-1 (HIF-1) transcriptional program is a frequent event in NETs, driving the production of large amounts of proangiogenic molecules such as vascular endothelial growth factor (VEGF), platelet-derived growth factor (PDGF), fibroblast growth factor (FGF), semaphorins and angiopoietins (8).

Clinical strategies encompassing angiogenesis inhibition have a definite place in the therapeutic armamentarium against NETs. The oral tyrosine kinase inhibitor (TKI) sunitinib is currently approved for pancreatic NETs (panNETs) (9), and a variety of new antiangiogenic agents are presently under clinical scrutiny for both GEP and bronchopulmonary (BP) NETs (10). In this review, we provide an overview of the current understanding of the molecular events driving neoangiogenesis in NENs, also discussing present and future therapeutic applications of antiangiogenic agents in the clinical arena.

## Angiogenesis in NETs

Tumor angiogenesis is a complex process through which a neoplasm creates its own vascularization, essential for obtaining the oxygen and nutrients necessary to grow beyond a certain, and well defined, volume. Moreover, this vascularization provides an access to the bloodstream that the tumor uses to metastasize. This is true for almost all malignancies, including NETs (7). Angiogenesis is tightly regulated by a complex balance between pro- and anti-angiogenic molecules, and a cross-talk exists between endothelial cells, pericytes and tumor cells. Indeed, while anti-apoptotic factors supporting the tumor growth are released by activated endothelial cells of the newly formed vessels, pro-angiogenic molecules are produced in turn by tumor cells, thereby sustaining the so called “angiogenic switch” and engaging neovascularization (11). In this context, pericytes can stimulate an autocrine VEGF-mediated prosurvival signaling in endothelial cells, further promoting neovascular sprouting and, indirectly, tumor growth (12). Influenced by the same family of molecular cues driving angiogenesis, tumor lymphangiogenesis has also a key role in metastasis formation, and possibly resistance to antiangiogenic therapy. In this context, VEGFs other than VEGF-A have been described to mediate the outgrowth of lymphatic vessels in NETs thereby leading to progression to stages of greater malignancy (13–15).

The vascular alterations observed in NETs are both quantitative and qualitative. Extensive neovascularization in the presence of low endothelial proliferation is indeed a hallmark of well-differentiated NETs, while a lower intratumor microvascular density is typically observed in poorly differentiated carcinomas (8, 16). Such a phenomenon, named as “neuroendocrine paradox”, is possibly related to the capability of well-differentiated NET cells to retain their normal precursors’ ability to stimulate the formation of a dense vascular network, with the angiogenesis of poorly differentiated neoplasms being instead primarily dependent on proliferation-induced hypoxia. The newly formed blood vessels are structurally and functionally aberrant in NETs (7). In particular, endothelial cells appear to contain multiple fenestrations (which are also typical of normal endocrine glands) and trans-endothelial channels while basement membranes are discontinuous and lack pericyte coverage, thus resulting in increased interstitial fluid pressure, vessel tortuosity and leakiness, as well as frequent hemorrhage.

**Figure 1:**
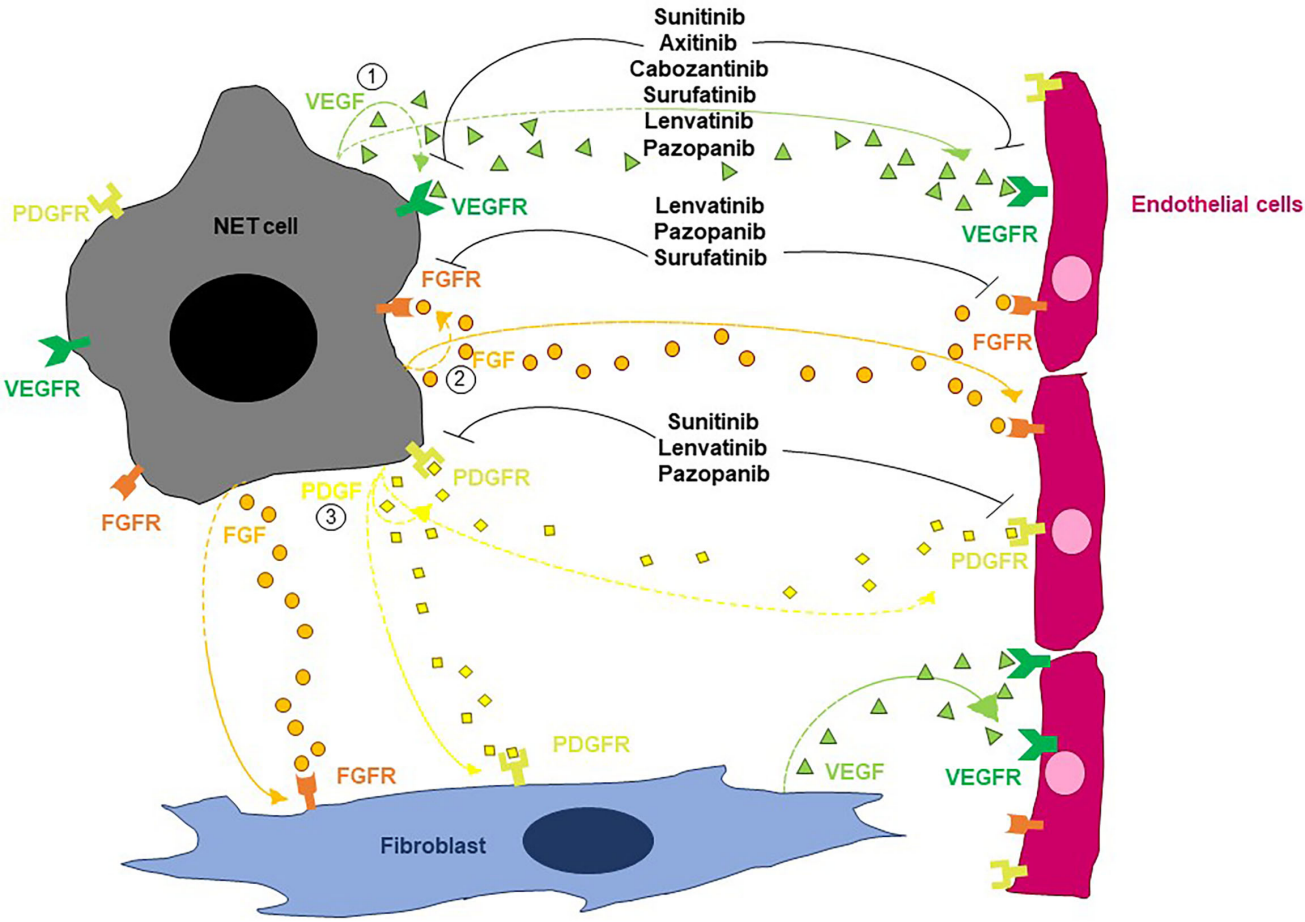
Schematic overview of the main pathways regulating angiogenesis in NETs. By acting on endothelial cells, VEGF stimulates both vascular endothelial mitogenesis and permeability. FGFs trigger endothelial cell migration, proliferation and differentiation as well as vessel formation. PDGF contributes to the angiogenic process by stimulating the recruitment of pericytes and the resulting vessel coverage. Multiple TKIs can interfere with the angiogenic process in NETs.

As depicted in **Figure 1**, three main molecular circuits regulate angiogenesis in NETs: the VEGF/VEGFR pathway, the FGF-dependent signaling and the PDGF/PDGFR axis (17). Vascular endothelial growth factor is constitutively expressed by normal neuroendocrine cells. Its expression is retained in up to 80% of GEP-NETs, where it drives angiogenesis through interaction with VEGFR-1 and VEGFR-2. The expression of VEGF is higher in well-differentiated malignancies with respect to poorly differentiated NENs, and parallels the expression of its receptors on both tumor and endothelial cells (8, 18–20). Tumor and stromal cells are not the only sources of VEGF in NETs, as tumor-infiltrating neutrophils have been shown to mobilize latent VEGF from the extracellular matrix through the release of metalloproteinase 9 (MMP-9), at least in mice (21). Mechanistically, VEGF acts in an autocrine or paracrine fashion triggering both vascular endothelial mitogenesis and permeability *via* activation of the Notch signaling in endothelial cells (18, 20, 22, 23). Evidence from the RIP1-Tag2 transgenic mouse model demonstrates that VEGF exerts a critical role throughout the whole course of the multistage process of pancreatic endocrine tumorigenesis (19). In particular, the selective knockout of *VEGF* in β cells of RIP1-Tag2 mice dampens both angiogenic switch and neovasculature formation in dysplastic islets, thus preventing the growth of panNETs (24). As in other cancers, the production of VEGF by NET cells is primarily regulated by local oxygen availability through the sensing activity of HIF-1 (23, 25). In this context, evidence demonstrates that panNETs arising in patients with Von-Hippel Lindau disease, a condition characterized by uncoupled oxygen levels/HIF-1 activity, show a distinct proangiogenic molecular signature when compared with sporadic panNETs, thus suggesting that different evolutionary trajectories are followed by these two entities (26).

A second angiogenic pathway modulating the progression of NETs involves FGF and its cognate receptors. The family of FGF is known to comprise 23 members (although there are only 18 FGFR ligands) and exerts multiple functions through the activation of FGFRs (27). This pathway has both direct and indirect effects on angiogenesis. Indeed, while directly stimulating endothelial cell migration, proliferation and differentiation as well as vessel formation and maturation, FGF also acts as a key regulator of proangiogenic molecules including VEGF and angiopoietins (28, 29). Fibroblast growth factor-1 and fibroblast growth factor-2 are expressed in approximately 40% and 100% of GEP-NENs respectively, while fibroblast growth factor receptor (FGFR) 1-4 are expressed by the 68-88% of these malignancies (30, 31). Fibroblast growth factor has a key role in maintaining tumor angiogenesis after an initiation phase primarily guided by the VEGF signaling, and the inhibition of the FGF/FGFR axis suppresses neoangiogenesis and tumor growth in the RIP1-Tag2 transgenic mouse model (32). Evidence demonstrates that FGF is a critical driver of VEGF-independent revascularization of panNETs and can therefore mediate evasive resistance to antioangiogenic therapy (33, 34).

The PDGF/PDGFR axis is another crucial mediator of NET progression. Platelet-derived growth factor contributes to the angiogenic process by stimulating the recruitment of pericytes and the resulting vessel coverage (35). Expression of PDGFR-α and PDGFR-β has been described in approximately 75% and 60% of GEP-NETs respectively (36, 37). In particular, while PDGFR-α is predominantly expressed by tumor cells, PDGFR-β is mainly expressed by pericytes and stromal cells. A positive association between PDGFR-α expression and tumor grade as well as between PDGFR-β expression and tumor microvascular density has been documented (36, 37), and the paracrine secretion of PDGF-DD by endothelial cells has been shown to stimulate NET proliferation (38). In this context, experiments in the RIP1-Tag2-PDGFD knockout model demonstrate that the disruption of the PDGF-DD signaling significantly delays panNET growth (7).

Mounting evidence indicates that semaphorins and angiopoietins contribute to neoangiogenesis in NETs. Semaphorins have shown both pro- and anti-angiogenic effects in NETs, and their activity is the result of the interaction with neuropilin and plexin receptors (7). Neuropilin receptors have been found in both pancreatic, intestinal and pulmonary NETs (39–41), while data on the expression of plexin receptors in NETs are currently lacking. Experiments in RIP1-Tag2 mice have shown that the expression of semaphorin 3A (SEMA3A) is progressively lost during tumor progression and that the inhibition of SEMA3A during the angiogenic switch may enhance tumor formation. Of note, re-expression of SEMA3A by viral gene transfer during late stages of pancreatic endocrine tumorigenesis leads to normalization of the tumor vasculature, increased pericyte coverage and inhibition of tumor progression (42, 43). Similar antiangiogenic effects have been also documented for SEMA3F in ileal NETs (44). On the other hand, protumorigenic activities have been attributed to SEMA4D and SEMA5A. In particular, inhibition of SEMA4D has been recently associated with impaired tumor growth *via* pericyte coverage alteration and vascular function modification in RIP1-Tag2 mice (45). SEMA5A can elicit angiogenesis, tumor growth, invasion and metastasis by activating c-met downstream its interaction with Plexin-B3 (46). Angiopoietins and angiopoietins receptors are widely expressed in NETs (47, 48). The overexpression of Angiopoietin-2 (Ang-2) in orthothopic NET xenografts in nude mice drives increased microvascular density and enhanced metastatic spread through lymphatic vessels (49). The blockade of the interaction between Ang-2 and its cognate receptor TIE2 determines regression of the tumor vasculature and inhibition of tumor progression in the RIP1-Tag2 mouse model of pancreatic endocrine cancerogenesis (50).

## Molecular mechanisms of resistance to antiangiogenic therapies in NETs

**Figure 2:**
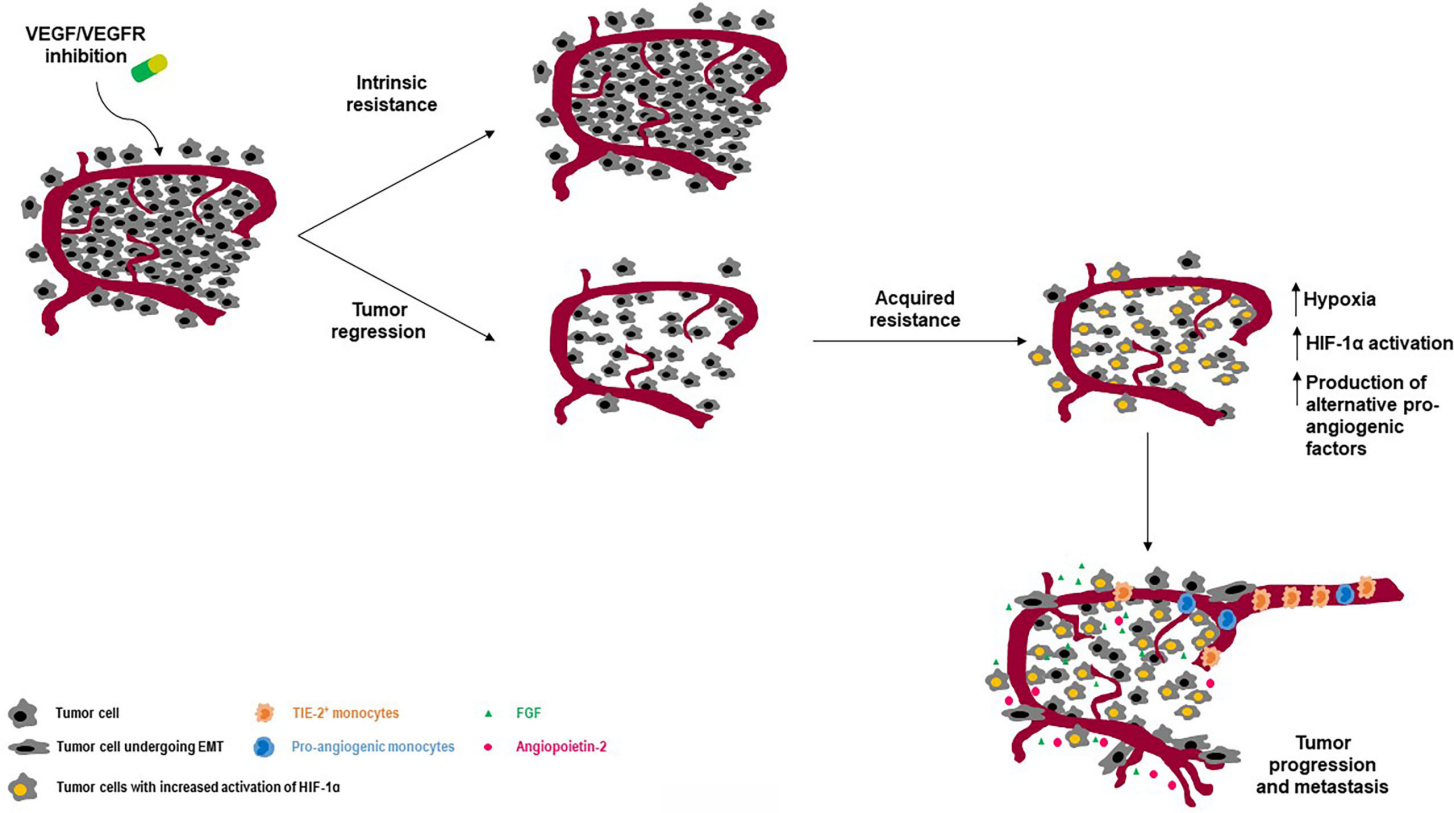
Intrinsic and acquired resistance to angiogenesis blockade: an overview on molecular determinants. While some tumors may show primary resistance to antiangiogenic agents, other may develop resistance upon blockade of the VEGF/VEGFR pathway. One of the main mechanisms leading to secondary resistance is the activation of HIF-1 as result of antiangiogenesis-induced hypoxia. Among other important events, there are the recruitment and/or activation of pro-angiogenic cells including TIE-2 expressing macrophages and the activation of the epithelial-to-mesenchymal transcriptional program in NEN cells.

Inhibition of angiogenesis has revealed therapeutic efficacy in NET patients. Nevertheless, resistance to antiangiogenic therapies inevitably occurs, and the biological events leading to such a phenomenon have been only partly elucidated. While primary resistance refers to an intrinsic unresponsiveness to antiangiogenic treatments, secondary (or acquired) resistance stems from tumor adaption to therapy, mostly as result of the activation of alternative proangiogenic circuits (51). **Figure 2** depicts the main biological events driving resistance to antiangiogenic therapies in NETs.

An established cause of resistance to antiangiogenic agents primarily acting through VEGF suppression is tumor hypoxia. Tumor hypoxia can stimulate HIF-1 activation, thus triggering neoangiogenesis through VEGF-independent mechanisms involving FGF, angiopoietins, and ephrins (33, 52). Evidence from the RIP1-Tag2 model demonstrates that inhibition of both VEGF and FGF signaling at the time of VEGF-independent tumor revascularization attenuates both revascularization and tumor growth (33). In this context, brivanib, a first-in-class, dual FGF-VEGF inhibitor has shown superior preclinical antitumor activity against panNETs when compared with single VEGF suppression or single FGF inhibition (34). A marked upregulation of Ang-2 and TIE2 has been observed in tumors from late-stage RIP1-Tag2 mice resistant to VEGFR blockade (53). In this context, dual Ang-2/VEGFR inhibition was shown to suppress tumor revascularization and progression, suggesting that the adaptive enforcement of Ang2-TIE2 signaling plays a key role in the establishment of evasive tumor resistance to anti-VEGF therapy. The upregulation of c-Met is another consequence of the chronic HIF-1 activation induced by tumor hypoxia. In RIP1-Tag2 mice, VEGFR blockade results in c-Met overexpression, leading to increased tumor growth, proliferation and invasion (54). Concurrent inhibition of VEGF and c-Met signaling is able to revert such effects *in vivo* (55).

The recruitment of bone marrow derived cells such as endothelial progenitor cells, pro-angiogenic monocytic cells or TIE2-expressing macrophages has been described in panNETs as a result of the hypoxic environment generated by VEGFR inhibition (13, 53). These cells promote the sprouting of new vessels, maintaining the high-demanded blood supply of tumor cells, while concurring to the generation of new premetastatic niches. A progressive increase in the number of tumor-associated macrophages has been described during the sequential progression from hyperplastic islets to angiogenic islets and ultimately invasive tumors in the RIP1-Tag2 model (56). A possible involvement of these cells in the establishment of adaptive resistance to VEGFR blockade through the exploitation of alternative proangiogenic pathways has been inferred.

An increased pericyte coverage has been detected in murine panNETs resistant to VEGFR2 inhibition as compared to those responsive to antiangiogenic therapy (57). Such a phenomenon has been related to a non-angiogenic mechanism of tumor vascularization named vascular co-option. When vascular co-option is activated, cancer cells grow around normal vessels pre-existing in the adjacent “normal” tissue, without the need of generating new vessels. Evidence demonstrates that this process contributes to the emergence of resistance to VEGFR inhibition in the RIP1-Tag2 model (33). Another non-angiogenic mechanism driving resistance to antiangiogenic therapy in panNETs is named “vascular mimicry”, and consists of cancer cells forming vascular channels to autonomously sustain their growth (58). An increased expression of Snail, vimentin and N-cadherin as well as a concurrent downregulation of E-cadherin has been observed in tumors treated with sunitinib, and the hypoxia-driven epithelial-to-mesenchymal transition can be therefore listed as an additional mechanism of resistance to angiogenesis blockade and tumor aggressiveness (43, 59).

Ion trapping and degradation of hydrophobic TKIs within the acidic lysosomal compartment is another mechanism leading to resistance to antiangiogenic therapy. Chloroquine, an agent able to permeabilize the lysosomal membrane, has been shown to enhance the antitumor activity of sunitinib in murine models of pancreatic endocrine carcinogenesis by stimulating the release of the TKI in the cytoplasm (60).

## Targeting angiogenesis in NETs: established and investigational agents

Proangiogenic pathways can be blocked at different levels in NETs. Both direct suppression of proangiogenic molecules such as VEGF and inhibition of receptors tyrosine kinase including VEGFR and FGFR through TKIs (i.e., sunitinib) or mAb (i.e., ramucirumab) have been exploited in clinical trials. **Table 1** provides an overview of the clinical investigations of antiangiogenic agents in patients with NETs. Being the targets of TKIs usually multiple, it is currently difficult to precisely determine to what extent their therapeutic effects are related to anti-angiogenesis, antiproliferative activity against tumor cells *per se*, or interference in the mechanisms of cross-talk between tumor cells and their microenvironment.

**Table 1:** An overview of completed studies with antiangiogenic agents in patients with NENs.

Study agent(s)	Main molecular targets	Study design and phase	Patient population	Enrolled patients	mPFS of the investigational agent/combination	Grade 3/4 AEs (frequency)
**Sunitinib** (9)	VEGFR-1,-2,-3, PDGFR	Double-blind, placebo-controlled, randomized phase 3 study	G1/G2 advanced progressive panNENs	171	11.1 months	Neutropenia (12%) Hypertension (10%) Palmar-plantar erythrodysesthesia Ashtenia (5%) Diarrhea (5%)
**Surufatinib** (61, 62)	VEGFR-1,-2,-3 FGFR-1 CSF-1R	Double-blind placebo- controlled, randomized phase 3 studies	Well-differentiated advanced progressive pancreatic (SANET-p) and extra pancreatic (SANET-ep) NENs	SANET-p trial: 172 SANET-ep trial: 198	SANET-p trial: 10.9 months SANET-ep trial: 9.2 months	SANET-p trial: Hypertension (38%) Proteinuria (10%) Hypertrigliceridemia (7%) SANET-ep trial: Hypertension (36%) Proteinuria (19%) Anemia (7%)
**Lenvatinib** (63)	VEGFR-1,-2,-3 FGFR-1,-2,-3,-4 PDGFRα c-KIT RET	Open-label phase 2 study	Advanced progressive panNENs and gastrointestinal NENs	PanNENs: 55 Gastrointestinal NENs: 56	panNENs: 15 months Gastrointestinal NENs: 15 months	Hypertension (22%) Fatigue (11%) Diarrhea (11%)
**Axitinib** (64)	VEGFR-1,-2,-3	Open-label phase 2 study	G1/G2 advanced progressive extrapancreatic NENs	30	27 months	Hypertension (63%)
**Axitinib + Octreotide LAR** (65)	VEGFR-1,-2,-3	Double-blind randomized phase 2/3 study	G1/G2 advanced progressive extrapancreatic NENs	256	17.2 months	Hypertension (21%) Cardiac disorders (3%) Fatigue (9%) Diarrhea (13%) Nausea (2%)
**Cabozantinib** (66)	MET VEGFR2 c-KIT RET AXL TIE2 FLT3	Open-label phase 2 study	Well-differentiated advanced progressive pancreatic and extrapancreatic NENs	PanNENs: 20 ExtrapancreaticNENs: 41	PanNENs: 21.8 months Extrapancreatic NENs: 31.4 months	Hypertension (13%) Hypophosphatemia (10%) Diarrhea (10%) Fatigue (5%)
**Pazopanib** (67)	VEGFR-1,-2,-3 FGFR-1,-3,-4 PDGFR-α, -β c-KIT	Randomized, placebo-controlled phase 2 study	Well-differentiated advanced progressive extrapancreatic NENs	171	12 months	Diarrhea (5%) Fatigue (8%) Nausea (5%) Hypertension (27%) Transaminase elevation (18%)
**Belzutifan** (68)	HIF-2α	Open-label phase 2 study	Advanced panNENs arising in the context of VHL syndrome	22	–	Anemia (8%)* Hypertension (8%)* Fatigue (5%)*
**Evofosfasmide + Sunitinib** (69)	DNA cross links VEGFR-1,-2,-3, PDGFR	Open-label, phase 2 study	Advanced progressive panNENs	17	10.4 months	Neutropenia (35%) Fatigue (18%) Thrombocytopenia (12%)
**Everolimus ± Bevacizumab** (70)	mTOR VEGF	Randomized phase 2 study	G1/G2 advanced progressive panNENs	150	16.7 months	Diarrhea (14%) Hyponatremia (12%) Hypophosphatemia (11%) Proteinuria (16%) Hypertension (41%)
**Atezolizumab + Bevacizumab** (71)	PD-L1 VEGF	Open-label, phase 2 study	G1/G2 advanced progressive pancreatic and extrapancreatic NENs	PanNENs: 20 Extrapancreatic NENs: 20	PanNENs: 14.9 months Extrapancreatic NENs: 14.2 months	Hypertension (20%) Proteinuria (8%)

*In the safety cohort (n=61 patients).

## VEGF/VEGFR-targeting agents

Bevacizumab is a mAb against VEGF and preliminary evidence suggested the antitumor activity of the drug in patients with NETs (69, 72, 73). However, no benefit in PFS was recorded in a randomized phase 3 trial comparing bevacizumab plus octreotide versus interferon plus octreotide in 427 patients who had high-risk NETs (74). In recent years, bevacizumab has been investigated in combination with chemotherapy, targeted agents or immunotherapy. In 2 separate phase 2 studies, bevacizumab has been tested in association with the capecitabine-oxaliplatin or FOLFOX regimens (75) in 40 and 36 patients with progressive NETs or NECs. Neither study met its primary endpoint, leading to objective responses in 18% and 25% of patients respectively. Another phase 2 trial investigated bevacizumab with 5-FU and streptozocin in 34 patients with progressive, well-differentiated panNETs. A median PFS of 23.7 months was observed, in the presence of an ORR of 56% (76). In a phase 2 trial, 150 patients with advanced panNETs were randomized to receive the mTOR inhibitor everolimus plus octreotide with or without bevacizumab (70). Of note, preclinical evidence suggests that the antitumor activity of mTOR inhibition in NETs results from a combination of antiproliferative and antiangiogenic effects (77). The treatment arm containing the antiangiogenic agent resulted in improved PFS compared to the control arm (16.7 months compared to 14 months; hazard ratio, 0.8; *p*=0.10), with objective responses seen in 31% and 12% of patients treated with or without bevacizumab respectively (*p*=0.005). Despite the encouraging efficacy outcomes, the higher rate of treatment-related toxicities observed in the investigational arm may limit further investigations on this combination. Similar results were achieved in a phase II study evaluating the combination of bevacizumab and sorafenib in 44 patients with advanced NETs (78). Despite a median PFS of 12.4 months, grade 3/4 toxicities were described in the 63% of the enrolled patients. Based on the evidence that anti-angiogenic agents such as bevacizumab may modulate the tumor immune microenvironment and decrease the expression of regulatory checkpoints on tumor-infiltrating lymphocytes (79), a single-arm, open-label, nonrandomized study has recently evaluated the association of bevacizumab with the anti-PD-L1 Ab atezolizumab in patients with advanced, progressive, well-differentiated NETs (71). Overall, 20 patients with panNETs and 20 patients with extra-pancreatic NETs have been enrolled, and an ORR of 20% and 15% has been recorded in the two cohorts. A median PFS of 14.9 months and 14.2 months has been reported in the pancreatic and extra-pancreatic cohort, suggesting the potential efficacy of this combination. Hypertension and proteinuria have been described as the most common treatment-emergent toxicities. Bevacizumab is currently being investigated in combination with chemotherapy or chemo-immunotherapy in multiple trials of patients with extrapulmonary NECs (80).

Ramucirumab, a humanized mAb targeting VEGFR2, has demonstrated preliminary evidence of efficacy when used in combination with chemotherapy in patients with gastric NEC (81). A prospective, multicenter, single-arm study is currently investigating ramucirumab plus dacarbazine in patients with advanced, progressive, well-differentiated panNETs (82).

The VEGF trap aflibercept has been investigated in a phase II, single-arm trial of 21 patients with advanced panNETs (83). An ORR of 9% has been reported, a finding consistent with other antiangiogenic agents in panNETs.

## Sunitinib

Sunitinib is the only antiangiogenic agent currently approved for the treatment of NETs. Sunitinib is an oral TKI targeting, among a number of different kinases, VEGFR-1, -2, -3 and PDFGR, and has demonstrated efficacy in the treatment of advanced, progressive panNETs. A double-blind, placebo-controlled, phase 3 study evaluated sunitinib 37.5 mg daily in 171 patients with low-to-intermediate grade, progressive panNETs (9). The trial demonstrated a statistically significant improvement in median PFS from 5.5 months on the placebo arm to 11.1 months on the sunitinib arm (hazard ratio, 0.42; *p*<0.001). A nonsignificant overall survival (OS) improvement of approximately 10 months was observed in the sunitinib arm compared with the placebo arm (84). Nausea, diarrhea, fatigue, cytopenia, hypertension and palmar-plantar erythrodydesthesia were the main treatment-related toxicities. A similar toxicity profile has been described in a recent phase IV study (85).

## Other multikinase inhibitors

Dual inhibitors of the VEGF/FGF signaling carry the promise of overcoming the mechanisms leading to adaptive resistance to sunitinib, and recent clinical research has focused on agents including surufatinib, lenvatinib, axitinib, cabozantinib and pazopanib. Surufatinib is an oral, selective inhibitor of VEGFR-1, -2, -3, FGFR-1 and colony stimulating factor-1 receptor (CSF-1R). The TKI has been tested at a dosage of 300 mg daily in a single-arm, multicenter, phase 1b/2 trial of 81 patients with low-to-intermediate grade advanced NETs (86). A median PFS of 21.2 months and 13.4 months was reported in 42 patients with panNETs and 39 patients with extrapancreatic NETs respectively. Two randomized, double-blind, placebo-controlled, phase 3 studies have recently investigated the safety and efficacy of surufatinib in Chinese patients with well-differentiated, progressive, advanced pancreatic (SANET-p trial) and extrapancreatic NETs (SANET-ep trial). The SANET-p trial (61) randomized 172 patients with panNETs to receive surufatinib or placebo in a 2:1 ratio. The investigator-assessed median PFS was 10.9 months for surufatinib versus 3.7 months for placebo (hazard ratio: 0.49; *p*=0.001), with an investigator-assessed overall response rate (ORR) of 19% in the investigational arm. The SANET-ep trial (62) randomized 198 patients with extrapancreatic NETs to receive surufatinib or placebo in a 2:1 ratio. The investigator-assessed median PFS was 9.2 and 3.8 months in the surufatinib and placebo arms respectively (hazard ratio: 0.33; *p*<0.0001). The ORR was 15%, and the majority of enrolled patients (84%) had G2 tumors. Overall, hypertension, proteinuria, hypertriglyceridemia and diarrhea were reported as the most frequent treatment-related grade 3 or worse adverse events. The occurrence of treatment-related adverse events including hypertension, proteinuria and hemorrhage in the first 4 weeks of treatment has been recently described to predict the antitumor efficacy of surufatinib (87). The efficacy and safety of surufatinib are being currently evaluated in two ongoing trials in the US (NCT02549937) and Europe (NCT04579679), and their results might lead to the approval of the drug in Western countries. It remains currently unclear whether surufatinib may be active in patients progressing to prior antiangiogenic therapy, and current investigations exclude from enrollment patients who have received prior VEGF/VEGFR targeted therapy.

Lenvatinib is an oral TKI targeting VEGFR-1, -2, -3, FGFR-1, -2, -3, -4, platelet-derived growth factor receptor α (PDGFRα), KIT and RET. The drug has been recently investigated in the phase 2 TALENT study at a dosage of 24 mg daily (63). A total of 55 patients with advanced panNETs and 56 patients with advanced gastrointestinal NETs have been enrolled. All patients had progressive disease according to RECIST criteria, and prior therapy with targeted agents was mandatory for inclusion in the panNET cohort. By central radiology review, the ORR was 44% and 16% for panNETs and gastrointestinal NETs respectively, and the median duration of response was 20 and 34 months in the two cohorts respectively. After a median follow-up of 23 months, the median PFS was 15 months for either panNETs and gastrointestinal NETs. Hypertension, fatigue and diarrhea were the most frequent G3/4 treatment-emergent adverse events. Dose reductions or interruptions were required in the 94% of patients. Although the ORR observed in the TALENT study is the highest reported to date with a TKI in advanced NETs, further clinical investigations of this agent in NETs have not been planned so far.

Axitinib is a TKI that selectively targets VEGFR-1, -2 and -3. In an open-label phase 2 study, axitinib 5 mg twice daily was investigated in 30 patients with progressive, advanced, low-to-intermediate grade NETs of extra-pancreatic origin (64). After a median follow-up of 29 months, a median PFS of 27 months was observed. Grade 3/4 hypertension was recorded in the 63% of the cohort, leading to treatment discontinuation in one fifth of enrolled patients. The double-blind, phase 2/3 AXINET trial has recently randomized 256 patients with advanced, low-to-intermediate grade, progressive, extra-pancreatic NETs to receive axitinib plus octreotide LAR or placebo plus octreotide LAR (65). Per blinded independent central review, the median PFS was 16.6 and 9.9 months in the axitinib and placebo arms respectively (HR: 0.69; *p*=0.01). An ORR of 13% and 3% has been reported in the investigational and control group respectively (*p*=0.004). Grade 3 or worse adverse events occurred in the 52% of the enrolled patients and included hypertension, cardiac disorders, fatigue, diarrhea and nausea/vomiting. One treatment-emergent death was reported in the axitinib arm. Overall, axitinib appears a promising candidate for future regulatory approval in patients with NETs.

Cabozantinib is an oral, potent inhibitor of MET, VEGFR2, KIT, RET, AXL, TIE2 and FLT3. The TKI has been tested at 60 mg daily in a two-cohort, phase 2 study enrolling 20 patients with panNETs and 41 patients with extra-pancreatic NETs (66). All patients had well-differentiated tumors and progressive disease according to RECIST 1.1 criteria. The ORR was 15% in either cohort, while a median PFS of 21.8 and 31.4 months was recorded in patients with pancreatic and extra-pancreatic neoplasms respectively. Hypertension, hypophosphatemia, diarrhea and fatigue were among the most common grade 3/4 adverse events. Dose reductions were required in the 80% of patients. The phase 3 CABINET trial (NCT03375320) is currently randomizing patients with well-differentiated, advanced, progressive, pancreatic or extra-pancreatic NET to receive cabozantinib 60 mg daily or placebo. Combinations of cabozantinib plus temozolomide (NCT04893785), lanreotide (NCT04427787) or ^177^Lu-DOTATATE (NCT05249114) are presently under scrutiny in phase 2 studies.

Pazopanib is an oral TKI targeting VEGFR -1, -2, -3, FGFR-1, -3, -4, PDGFR-α and -β and c-KIT. The drug has been investigated in the open-label, phase 2 PAZONET trial (88). In 44 patients with advanced, progressive, well-differentiated NETs, the TKI was associated with a median PFS of 9 months. The most common grade 3/4 toxicities of pazopanib included diarrhea, fatigue and hypertension, and drug dosage reductions were required in approximately one fifth of enrolled patients. More recently, pazopanib has been tested at 800 mg daily in the phase 2 Alliance A021202 study (67). The trial randomized 171 patients with well-differentiated, progressive, extrapancreatic NETs to receive pazopanib or placebo. After a median follow-up of 31 months, a median PFS of 12 and 8 months was recorded in patients treated with pazopanib or placebo respectively (HR: 0.53; *p*=0.0005). Pazopanib was associated with an ORR of only 2%. Among pazopanib-treated patients, treatment-related grade 3/4 adverse events were reported in 61% of cases, and hypertension, fatigue, nausea, diarrhea and transaminases elevation were the most common toxicities. Pazopanib has also demonstrated clinical activity against panNETs arising in the context of von Hippel-Lindau syndrome. In a single-arm study enrolling 31 patients with this inherited syndrome, the drug induced objective responses in 53% of 17 pancreatic lesions (89).

Nintedanib is an oral inhibitor of FGFR1-3, VEGFR1-3 and PDGFR. The drug has been tested in a phase II study of 32 patients with extra-pancreatic NETs on a stable dose of somatostatin analog (90). A median PFS of 11 months has been observed, and toxicities were manageable.

## HIF inhibitors and hypoxia-activated prodrugs

Novel antiangiogenic compounds investigated in patients with NETs comprise the HIF-2α inhibitor belzutifan and the hypoxia-activated prodrug evofosfamide. Belzutifan has been recently tested at 120 mg daily in an open-label, phase 2 trial of 61 patients with von Hippel-Lindau syndrome. Among 22 patients harboring a panNET, objective responses were seen in 90% of cases, with complete responses in 14% of the cohort (68). Anemia and fatigue were the most common adverse events, being reported in 90% and 66% of patients respectively. On this basis, belzutifan has received regulatory approval for the treatment of tumors arising in the context of Von-Hippel Lindau syndrome. An international phase 2 study of belzutifan in patients with sporadic panNETs is currently ongoing (NCT04924075). Evofosfamide is a prodrug of the alkylating agent bromoisophosphoramide mustard. The release of the active drug occurs exclusively under hypoxic conditions, and results in intra- and inter-strand DNA cross links in tumor cells. Given the well-known ability of sunitinib in inducing intratumor hypoxia, evofosfamide has been recently investigated in combination with sunitinib in the open-label, Simon’s two-stage design, phase II SUNEVO trial (69). The study enrolled 17 patients with advanced panNETs, and only prior therapy with somatostatin analogs was permitted. After a median follow-up of 16 months, three objective responses were recorded, in the presence of a median PFS of 10.4 months. Grade 3 or worse treatment-related adverse events were reported in the 65% of the cohort, the most frequent being neutropenia, fatigue and thrombocytopenia. Overall, treatment discontinuation due to toxicity was required in 88% of the patients. In light of the unfavorable safety profile and the modest efficacy shown by sunitinib and evofosfamide in this study, further clinical investigations of this combination have not been planned.

## Future directions for angiogenesis blockade in NETs

Developing new antiangiogenic agents, testing new combinations of antiangiogenic agents with targeted drugs or immunotherapy and defining the correct positioning of antiangiogenic therapies in the context of treatment sequences are among the main priorities for future research on angiogenesis blockade in NETs.

Angiogenesis is a complex process involving distinct biological mechanisms. Mechanistically, endothelial cell proliferation, vessel guidance, vessel maturation, stabilization and quiescence are driven by different families of molecular cues, and angiogenic processes can be thereby inhibited at different levels. There is a need to identify and characterize additional molecular regulators of angiogenesis in NETs in order to develop the next generation of antiangiogenic drugs to be tested (alone or in combination) in clinical trials. Moreover, since evidence demonstrates that different angiogenic molecules may be expressed differently during tumor progression, a precise understanding of the molecular events driving neoangiogenesis during NET evolution might be instrumental to provide molecular-level guidance on the correct positioning of angiogenesis blockade throughout the treatment journey of NET patients. Combinatorial strategies aimed at concurrently disrupting key pathways operating in NET progression (i.e., concurrent inhibition of angiogenesis and mTOR signaling) have been only partially investigated. In a phase II study of bevacizumab and temsirolimus, an ORR of 41% and a median PFS of 13.2 months were observed among 58 patients with panNETs, in the presence of toxicities leading to treatment discontinuation in approximately one third of the cohort (91). While the efficacy/toxicity ratio of combinatorial treatments should be always carefully scrutinized in relatively indolent tumors such as NETs, clinical trials should explore the impact of targeted agent combinations in disease settings where tumor shrinkage is the goal of treatment (i.e., neoadjuvant setting). Accumulating evidence indicates that a tight link exists between aberrant tumor angiogenesis and the immune microenvironment, and antiangiogenic agents have been shown to synergize with immune checkpoint inhibitors in malignancies including renal cell carcinoma, endometrial cancer and hepatocellular carcinoma (92). Future studies should assess the potential of antiangiogenic agents in tuning the microenvironment of NETs from an immune-suppressive to an immune-supportive one, thus enhancing the efficacy of immunotherapy.

## Conclusions

The concept of angiogenesis inhibition as a potential weapon against cancer was first proposed by Folkman in the 70s (93). After the initial skepticism of the scientific community, multiple lines of evidence have demonstrated that antiangiogenic agents can be clinically effective in controlling tumor growth. Sunitinib is the only antiangiogenic drug approved for the treatment of NETs, and its use is restricted to pancreatic primaries. Newer TKIs including surufatinib, cabozantinib, axitinib and lenvatinib seem to possess more potent antitumor activity, probably as result of their multi-targeting potential, and might be utilized as monotherapy or as backbone for combinatorial treatment of both pancreatic and extra-pancreatic NETs in the near future. While it is currently unclear whether combinations of antiangiogenic agents with chemotherapeutics, targeted agents or immunotherapy are more effective than the monotherapy, the results of the first study comparing the efficacy of sunitinib versus PRRT in patients with progressive panNETs are awaited soon (NCT02230176). In the absence of predictors of response and given the lack of high-level evidence on optimal treatment sequences, clinical wisdom continues to be critical in defining the timing of antiangiogenic therapy in patients with NETs.

## Author contributions

All authors contributed to literature review and to the writing the manuscript. All authors approved the final version of the manuscript.

## Funding

This work was supported by the Associazione Italiana per la Ricerca sul Cancro [MFAG #23583] and Associazione per la Ricerca Biomolecolare Onlus, Acquaviva, Italy [2020].

## Conflict of interest

The authors declare that the research was conducted in the absence of any commercial or financial relationships that could be construed as a potential conflict of interest.

## Publisher’s note

All claims expressed in this article are solely those of the authors and do not necessarily represent those of their affiliated organizations, or those of the publisher, the editors and the reviewers. Any product that may be evaluated in this article, or claim that may be made by its manufacturer, is not guaranteed or endorsed by the publisher.
